# Effectiveness and safety of Baduanjin for schizophrenia: A protocol for systematic review and meta-analysis

**DOI:** 10.1097/MD.0000000000032007

**Published:** 2022-12-02

**Authors:** Haiyuan Wu, Kaiyuan Xue, Aineng Peng, Jianbo Chai, Yonghou Zhao

**Affiliations:** a Heilongjiang University of Traditional Chinese Medicine, Harbin, Heilongjiang Province, China; b Department of Neurology, Heilongjiang Mental Hospital, Harbin, Heilongjiang Province, China.

**Keywords:** Baduanjin, meta-analysis, schizophrenia, systematic review

## Abstract

The cause of schizophrenia is still unknown, the course of the disease is long and its onset is thought to be related to neurodevelopmental, genetic, and oxidative stress factors and so on. There is no means of cure. Typical drug therapy is effective in treating the acute stage of schizophrenia, while the impaired social and life functions of patients are often neglected. Baduanjin is a traditional Chinese physical and breathing exercise that not only strengthens the muscles, and moves the joints, but also exercises the will. Many studies have been reported in the study on the application of Baduanjin to schizophrenic patients to promote recovery, but no research systematically evaluates the therapeutic effects and safety of Baduanjin for schizophrenic patients. This study aims to systematically investigate the efficacy and safety of Baduanjin in the treatment of schizophrenic patients.

**Methods:**

Reports of randomized controlled trials (RCTs) on Baduanjin for schizophrenia will be searched in the following data sources, including 3 English databases（PubMed, EMBASE, Cochrane Library）and 4 Chinese databases（China National Knowledge Infrastructure, Chinese Biomedical Literature, Wanfang, and China Clinical Trials Registry Database）, and their publication time is restricted from the establishment of the database to October 1, 2022. Two reviewers will independently perform study selection, data extraction, and quality assessment. RevMan V.5.4 software will be used for meta-analysis. The protocol will be performed according to preferred reporting items for systematic reviews and meta-analysis protocols (PRISMA-P) guidelines.

**Results:**

The results will provide a systematic overview of the current evidence on the use of Baduanjin to treat schizophrenia.

**Conclusion:**

The conclusions of this study will help clarify whether Baduanjin is effective and safe for treating schizophrenia.

## 1. Introduction

Schizophrenia is a serious mental illness with an unknown etiology that requires long-term treatment and is characterized by a high relapse rate and high disability rate. According to the Global Burden of Disease Study, the global prevalence of schizophrenia ranges from 0.33% to 0.75%.^[1]^ It often strikes in youth or adulthood and has a long course.^[2,3]^ It ranks 16th in disability-adjusted life years (DALYs) and has become a major public health problem.^[4]^ The burden of schizophrenia is increasing with population growth and aging.^[5]^ Its treatment consists mainly of pharmacotherapy and psychotherapy.^[6,7]^ Notably, patients with schizophrenia tend to overlook deficits in somatic functioning, such as poorer than normal static and dynamic balance.^[8]^ Exercise, as a non-pharmacological intervention, can improve social and cognitive functioning by improving lifestyles, and may reduce their risk of cardiovascular diabetes, obesity, and other diseases.^[9–13]^ Baduanjin exercises not only improve motor coordination but also promote interpersonal interactions and improve functional impairment.^[14]^ However, there are no convincing systematic reviews and meta-analyses to evaluate the effects of Baduanjin on schizophrenia, hence we designed this study to better understand the efficacy and safety of Baduanjin in the treatment of schizophrenia and to provide a more reliable evidence-based basis for its treatment of schizophrenia.

## 2. Methods and analysis

### 2.1. Study registration

This study has been registered on PROSPERO（CRD42022369712）. We will adhere to the recommendations outlined in Preferred Reporting Items For Systematic Review and Meta-Analysis Protocols (PRISMA-P) 2015 statement.^[15]^

### 2.2. Eligibility criteria

#### 2.2..1. Types of studies.

RCTs of Baduanjin for schizophrenia will be included.

#### 2.2.2. Types of participants.

The subjects must meet the diagnosis criteria for schizophrenia including the American Diagnostic and Statistical Manual of Mental Disorders (DSM-V) or the Chinese Classification and Diagnostic Criteria for Mental Disorders or other criteria.^[16,17]^ No restriction on their age, race, and country.

#### 2.2.3. Types of interventions.

The experimental group will include patients treated with Baduanjin, including different genres of Baduanjin. The control group will include patients who receive Western medicine or Chinese herbal medicine, with the exception of Baduanjin.

#### 2.2.4. Types of outcome measures.

The main outcomes include changes in patient mental status, psychological status, and behavior scores, and specific aspects such as socialization.^[16,18,19]^ The additional outcomes include changes in individual satisfaction and quality of life, as well as adverse events.

### 2.3. Search strategy

From the establishment of the databases to October 1, 2022, we will search RCTs of Baduanjin for the treatment of schizophrenia, and data sources include 3 English databases（PubMed, EMBASE, Cochrane Library）and 4 Chinese databases（China National Knowledge Infrastructure, Chinese Biomedical Literature, Wanfang, and China Clinical Trials Registry Database）. In addition, a manual search of the reference lists of relevant research articles will be conducted. The search strategy for PubMed is shown in Table 1.

**Table 1:** Search strategy for PubMed.

Number	Search terms
#1	Baduanjin [Title/Abstract]
#2	Baduan jin [Title/Abstract]
#3	Eight section brocades [Title/Abstract]
#4	Eight-section Brocade [Title/Abstract]
#5	Eight trigrams boxing [Title/Abstract]
#6	Eight-treasured exercises [Title/Abstract]
#7	Eight pieces of brocade [Title/Abstract]
#8	Eight Brocade Section [Title/Abstract]
#9	Baduanjin exercises [Title/Abstract]
#10	Baduanjin exercise [Title/Abstract]
#11	Baduanjin Qigong [Title/Abstract]
#12	#1 OR #2 OR #3 OR #4 OR #5 OR #6 OR #7 OR #8 OR #9 OR #10 OR #11
#13	Schizophrenia, schizophrenias [Mesh]
#14	Schizophrenic Disorders [Mesh]
#15	Schizophrenic Disorder [Mesh]
#16	Disorders, Schizophrenic [Mesh]
#17	Disorder, Schizophrenic [Mesh]
#18	#13 OR #14 OR #15 OR #16 OR #17
#19	randomized controlled trial [Title/Abstract]
#20	clinical trial randomized [Title/Abstract]
#21	controlled clinical trial [Title/Abstract]
#22	clinical trial [Title/Abstract]
#23	randomized [Title/Abstract]
#24	randomly [Title/Abstract]
#25	trial [Title/Abstract]
#26	#19 OR #20 OR #21 OR #22 OR #23 OR #24 OR #25
#27	#12 AND #18 AND #26

### 2.4. Data selection and extraction

#### 2.4.1. Study selection.

**Figure 1.**
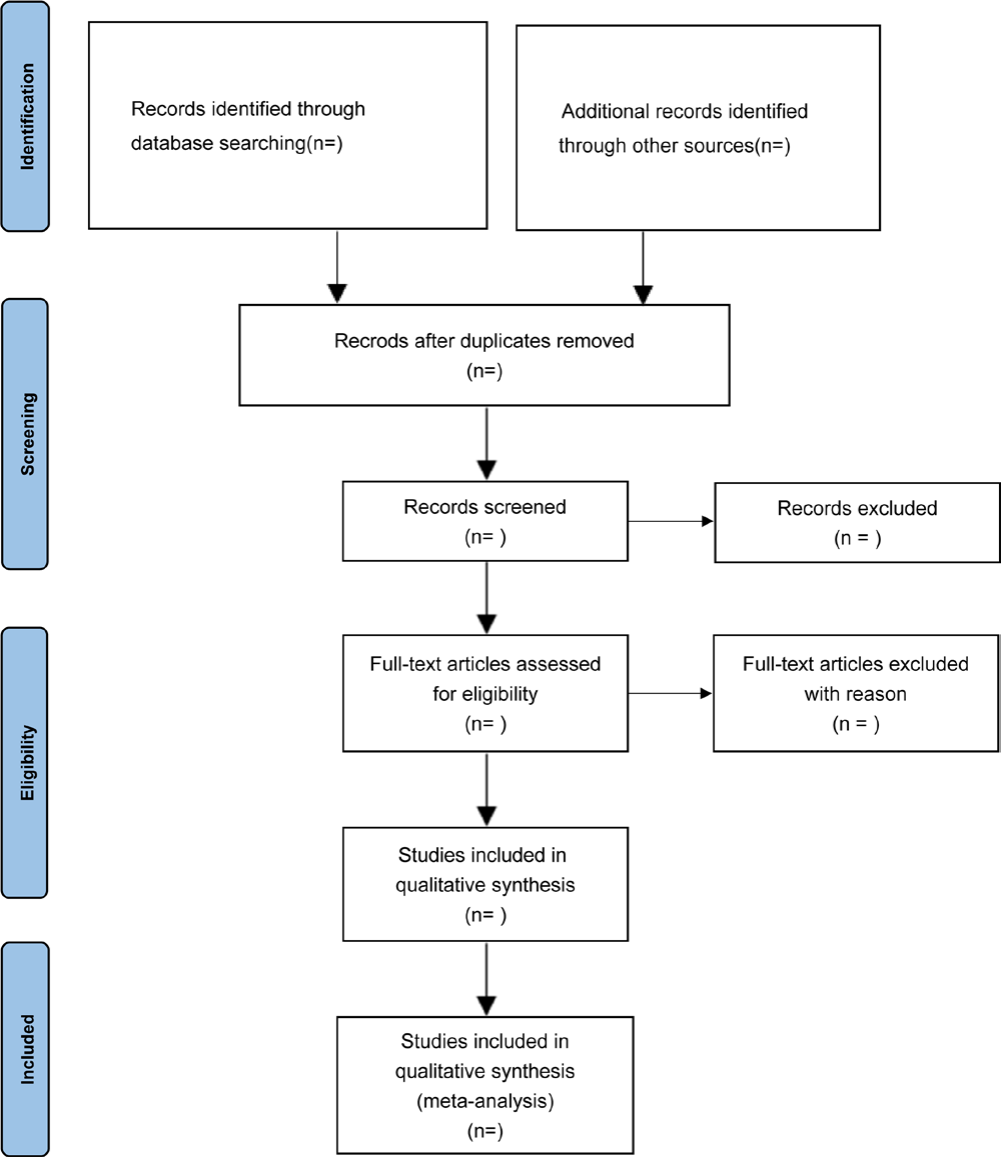
Flow chart of study selection.

Two investigators will review the completed articles that meet the inclusion and exclusion criteria, perform a secondary screening of abstracts and conclusions, and ultimately screen all eligible articles by reading the full text. If there is any disagreement in this process, a third study team member will be asked to assist in determining this. The entire study selection process is summarized in the flowchart in Figure 1.

#### 2.4.2. Data extraction and management.

Two researchers will independently extract data from the included studies, which will include information about the studies (year of publication, first author, sample size, age and sex of participants, duration of disease, interventions, primary and secondary outcomes, effectiveness, and adverse events). If there is any disagreement between the 2 reviewers during the screening process, they will consult a third reviewer to make a final decision.

#### 2.4.3. Assessment of the risk of bias and quality of the included studies.

The methodological quality of the investigation will be assessed on the basis of 7 items by applying the Cochrane systematic evaluation tool.^[20]^ The risk of bias for each item will be assessed and the results will be classified as low risk, unclear risk, and high risk.

#### 2.4.4. Measures of treatment effect.

Continuous outcome data will be presented as mean differences with 95% confidence intervals.

#### 2.4.5. Dealing with missing data.

If possible, we will try to contact the corresponding author for complete research or relevant data. If the author cannot be contacted, the available data will be analyzed, and the study will be excluded from the analysis.

#### 2.4.6. Data synthesis.

We will use RevMan V.5.4 for meta-analysis. Continuous outcome data will be presented as mean differences with 95% confidence intervals. We will determine whether to use a fixed-effects or random-effects model based on the results of the heterogeneity of research statistics, and we will use descriptive analysis when quantitative analysis is not appropriate.

#### 2.4.7. Subgroup analysis.

If there exists significant heterogeneity, we will analyze the age, gender, duration of illness, and the types of Baduanjin used by the schizophrenic patients participating in the study.

#### 2.4..8. Sensitivity analysis.

We will perform sensitivity analysis to improve the stability of the results.

#### 2.4.9. Quality of evidence.

We will use the Grading of Recommendations Assessment, Development, and Evaluation (GRADE) to classify the quality of evidence into 4 levels: very low, low, medium, or high.^[21]^

## 3. Discussion

First-generation and second-generation anti-schizophrenic drugs mainly target biochemical changes in the brain of schizophrenic patients, but are ineffective in alleviating negative symptoms such as cognitive and hippocampal deficits primarily target biochemical alterations in the patient’s brain.^[22]^ Negative symptoms and cognitive dysfunction in patients with schizophrenia are strongly associated with their quality of life, social functioning, and poor prognosis.^[23]^ Exercise therapy has been shown to improve patients’ positive and negative symptoms, quality of life, cognition, and hippocampal plasticity, and has been proposed as a primary non-pharmacological treatment modality that can improve patients’ quality of life and ability to return to society and family.^[24–28]^ Baduanjin is one of the traditional Chinese guided exercises dating back to the 12th century, and has been shown to improve balance in stroke, Parkinson disease, and frail elderly.^[29,30]^ Compared to ordinary aerobic exercises, Baduanjin is slower, gentler, more consistent, more varied, and more focused on mind-body integration exercises. It is mainly characterized by regular breathing rhythms and slow technical movements and can be used as an exercise therapy for mental conditioning.^[31]^ It does not require expensive fees and can be performed at any suitable place during leisure time. From the existing studies, we are unable to make evidence-based extrapolations for the treatment of schizophrenia with Baduanjin, and this is the first meta-analysis to apply Baduanjin exercise in the treatment of schizophrenia, with the aim of clarifying the efficacy and safety of Baduanjin in the treatment of schizophrenia, and the results of this study may provide a scientific basis for the clinical treatment of the disease.

## Author contributions

**Conceptualization**: Haiyuan Wu, Yonghou Zhao.

**Data curation**: Kaiyuan Xue, Aineng Peng.

**Formal analysis**: Kaiyuan Xue, Haiyuan Wu.

**Methodology**: Jianbo Chai.

**Project administration**: Yonghou Zhao.

**Writing—original draft**: Haiyuan Wu.

**Writing—review & editing**: Kaiyuan Xue.

## References

[1] Moreno-Küstner B Martín C Pastor L. Prevalence of psychotic disorders and its association with methodological issues. A systematic review and meta-analyses. PLoS One. 2018;13:e0195687. 29649252 10.1371/journal.pone.0195687 PMC5896987

[2] Jauhar S Johnstone M McKenna PJ. Schizophrenia. Lancet. 2022;399:473–86. 35093231 10.1016/S0140-6736(21)01730-X

[3] Harvey PD Green MF Keefe RSE. Cognitive functioning in schizophrenia: a consensus statement on its role in the definition and evaluation of effective treatments for the illness. J Clin Psychiatry. 2004;65:361–72. 15096076

[4] Charlson FJ Ferrari AJ Santomauro DF. Global epidemiology and burden of schizophrenia: findings from the global burden of disease study 2016. Schizophr Bull. 2018;44:1195–203. 29762765 10.1093/schbul/sby058 PMC6192504

[5] Larroumets P. Schizophrenia: study of epidemiological and economic regional disparities. Value Health. 2018;21:S76.

[6] Remington G Addington D Honer W. Guidelines for the pharmacotherapy of schizophrenia in adults. Can J Psychiatry. 2017;62:604–16. 28703015 10.1177/0706743717720448 PMC5593252

[7] Maroto Martin L Hervías Higueras P. Psychosocial therapy in schizophrenia. Eur Psychiatry. 2016;33:S566–S566.

[8] Aso K Okamura H. Association between falls and balance among inpatients with schizophrenia: a preliminary prospective cohort study. Psychiatr Q. 2019;90:111–6. 30328019 10.1007/s11126-018-9609-0

[9] McNamee L Mead G MacGillivray S. Schizophrenia, poor physical health and physical activity: evidence-based interventions are required to reduce major health inequalities. Br J Psychiatry. 2013;203:239–41. 24085733 10.1192/bjp.bp.112.125070

[10] Biondo J Bryl K. S190. When words aren’t enough: dance/movement therapy and schizophrenia. Schizophr Bull. 2020;46:S110–1.

[11] Falkai P Malchow B Schmitt A. Aerobic exercise and its effects on cognition in schizophrenia. Curr Opin Psychiatry. 2017;30:171–5. 28230631 10.1097/YCO.0000000000000326

[12] Scheewe TW Takken T Kahn RS. Effects of exercise therapy on cardiorespiratory fitness in patients with schizophrenia. Med Sci Sports Exerc. 2012;44:1834–42. 22525773 10.1249/MSS.0b013e318258e120

[13] Suvisaari J Keinänen J Eskelinen S. Diabetes and schizophrenia. Curr Diab Rep. 2016;16:16. 26803652 10.1007/s11892-015-0704-4

[14] Ho R Lo P Au Yeung FE. 0619 - Tai-chi exercises for schizophrenia. Eur Psychiatry. 2014;29:1.24119631

[15] PRISMA-P G Moher D Shamseer L. Preferred reporting items for systematic review and meta-analysis protocols (PRISMA-P) 2015 statement. Syst Rev. 2015;4:1. 25554246 10.1186/2046-4053-4-1 PMC4320440

[16] Kay SR Fiszbein A Opler LA. The positive and negative syndrome scale (PANSS) for schizophrenia. Schizophr Bull. 1987;13:261–76. 3616518 10.1093/schbul/13.2.261

[17] Dai Y Yu X Xiao Z. Comparison of Chinese and international psychiatrists’ views on classification of mental disorders. Asia Pac Psych. 2014;6:267–73. 10.1111/appy.12146 25139538

[18] Overall JE Gorham DR. The brief psychiatric rating scale. Psychol Rep. 1962;10:799–812.

[19] Behere R Raghunandan V Venkatasubramanian G. TRENDS- A tool for recognition of emotions in neuropsychiatric disorders. Indian J Psychol Med. 2008;30:32.

[20] Cumpston M Li T Page MJ. Updated guidance for trusted systematic reviews: a new edition of the Cochrane Handbook for Systematic Reviews of Interventions. Cochrane Editorial Unit, ed. Cochrane Database of Systematic Reviews. 2019. 10.1002/14651858.ED000142 PMC10284251 31643080

[21] Balshem H Helfand M Schünemann HJ. GRADE guidelines: 3. Rating the quality of evidence. J Clin Epidemiol. 2011;64:401–6. 21208779 10.1016/j.jclinepi.2010.07.015

[22] Girdler SJ Confino JE Woesner ME. Exercise as a treatment for schizophrenia: a review. Psychopharmacol Bull. 2019;49:56–69. 30858639 10.64719/pb.4584 PMC6386427

[23] Marder SR Cannon TD. Schizophrenia. Ropper AH, ed. N Engl J Med. 2019;381:1753–61. 31665579 10.1056/NEJMra1808803

[24] Scheewe TW Backx FJG Takken T. Exercise therapy improves mental and physical health in schizophrenia: a randomised controlled trial. Acta Psychiatr Scand. 2013;127:464–73. 23106093 10.1111/acps.12029

[25] Yuen M Ouyang HX Miller T. Baduanjin Qigong improves balance, leg strength, and mobility in individuals with chronic stroke: a randomized controlled study. Neurorehabil Neural Repair. 2021;35:444–56. 33825587 10.1177/15459683211005020

[26] Kong L Herold CJ Cheung EFC. Neurological soft signs and brain network abnormalities in schizophrenia. Schizophr Bull. 2020;46:562–71. 31773162 10.1093/schbul/sbz118 PMC7147582

[27] Wolf RC Rashidi M Schmitgen MM. Neurological soft signs predict auditory verbal hallucinations in patients with schizophrenia. Schizophr Bull. 2021;47:433–43. 33097950 10.1093/schbul/sbaa146 PMC7965075

[28] Rathod B Kaur A Basavanagowda DM. Neurological soft signs and brain abnormalities in schizophrenia: a literature review. Cureus. 2020;12:e11050. 33224647 10.7759/cureus.11050 PMC7676438

[29] Bao X Xiang QQ Shao YJ. Effect of sitting Ba-Duan-Jin exercises on balance and quality of life among older adults: a preliminary study. Rehabil Nurs. 2020;45:271–8. 30730382 10.1097/rnj.0000000000000219

[30] Liu X Seah JWT Pang BWJ. A single-arm feasibility study of community-delivered Baduanjin (Qigong practice of the eight Brocades) training for frail older adults. Pilot Feasibility Stud. 2020;6:105. 32699644 10.1186/s40814-020-00649-3 PMC7372818

[31] Zhang P Li Z Yang Q. Effects of Taijiquan and Qigong exercises on depression and anxiety levels in patients with substance use disorders: a systematic review and meta-analysis. Sports Med Health Sci. 2022;4:85–94. 35782275 10.1016/j.smhs.2021.12.004 PMC9219269

